# Hospitalization Events among Children and Adolescents with Sickle Cell Disease in Basra, Iraq

**DOI:** 10.1155/2015/195469

**Published:** 2015-10-26

**Authors:** Zeina A. Salman, Meaad K. Hassan

**Affiliations:** ^1^Center for Hereditary Blood Diseases, Basra Maternity and Children Hospital, Basra, Iraq; ^2^Department of Pediatrics, College of Medicine, University of Basra, Basra, Iraq

## Abstract

*Objectives*. Despite improvements in the management of sickle cell disease (SCD), many patients still experience disease-related complications requiring hospitalizations. The objectives of this study were to identify causes of hospitalization among these patients and factors associated with the length of hospital stay (LOS) and readmission. *Methods*. Data from 160 patients (<14 years old) with SCD who were admitted to the Basra Maternity and Children's Hospital from the first of January 2012 through July 2012 were analyzed. *Results*. The main causes of hospitalization were acute painful crises (73.84%), infections (9.28%), acute chest syndrome (8.02%), and acute splenic sequestration crisis (6.32%). The mean LOS was 4.34 ± 2.85 days. The LOS for patients on hydroxyurea (3.41 ± 2.64 days) was shorter than that for patients who were not (4.59 ± 2.86 days), *P* < 0.05. The readmission rate (23.1%) was significantly higher among patients with frequent hospitalizations in the previous year (OR 9.352, 95% CI 2.011–43.49), asthma symptoms (OR 4.225, 95% CI 1.125–15.862), and opioid use (OR 6.588, 95% CI 1.104–30.336). Patients on hydroxyurea were less likely to be readmitted (OR 0.082, 95% CI 0.10–0.663). *Conclusions*. There is a relatively high readmission rate among patients with SCD in Basra. The use of hydroxyurea significantly decreases the LOS and readmission rate.

## 1. Introduction

Sickle cell disease (SCD) is a multisystem disease associated with episodes of acute illness and progressive organ damage, and it represents a major public health problem because of its associated morbidity and mortality [1]. The prevalence of sickle hemoglobin (Hb S) in Basra is 6.48%, with a gene frequency of 0.0324% [2].

Patients with SCD have a chronic hemolytic anemia and can suffer from sudden, severe, and life-threatening complications caused by the acute sickling of red blood cells, with resultant pain or organ dysfunction. Repetitive sickling events can result in irreversible organ damage [3]. Children with SCD should be treated by experts, most often pediatric hematologists, for the management of this disease [4].

Comprehensive care and advances in clinical investigations have reduced the morbidity and mortality associated with SCD, especially in young children, and more patients are now surviving into adulthood. Most children with sickle cell anemia (93.9%) and nearly all children with milder forms of SCD (98.4%) now live to become adults [5, 6].

Severe complications of SCD often require hospitalization. The hospitalization of children with SCD constitutes a significant burden on their caregivers. The hospital admission pattern of children with SCD varies in different parts of the world.

Although acute, painful crises account for the majority of admissions in many countries. Infections are still the main cause of admissions in other areas, particularly in developing countries [7–10].

The average rate of painful crises prompting medical evaluation in sickle cell anemia (SCA) is 0.8 crises per year, although it is often treated inadequately in the emergency department [11, 12].

Approximately 40% of patients never seek medical attention for pain, while approximately 5% of patients account for one-third of all painful crises events requiring medical attention [11].

Acute chest syndrome (ACS), another important cause of morbidity and mortality among patients with SCD, refers to a constellation of findings that include a new radiodensity on chest radiograph, fever, respiratory distress, and pain that occurs often in the chest. Because of the clinical overlap between pneumonia and ACS, all episodes should be treated promptly with antimicrobial therapy including at least a macrolide and a 3rd-generation cephalosporin to treat the most common pathogens associated with ACS [4].

Many patients with SCD experience inpatient hospitalization for complications of the disease, with many who are also readmitted. Inpatient hospitalization for all children younger than 5 years was recommended by many centers because of the high risk of infection. In addition, all children, regardless of age, with the following high-risk features should be admitted: temperature above 38.5°C, marked lethargy, chest pain and/or shortness of breath, sudden onset of severe headache or seizures, sudden onset of pallor, abdominal distension, priapism, ill or toxic appearance, pain refractory to home treatment and joint pain, swelling, and redness [4, 11].

Identifying factors that can predict risk of readmission in SCD patients allows for the early diagnosis and treatment of groups particularly at risk, thus decreasing morbidity, improving quality of life, and reducing the SCD-related burden on health services [8].

The current study was conducted to identify the main causes of hospitalization among patients with SCD in Basra and to determine the factors associated with the length of hospitalization and with readmission of children and adolescents with SCD.

## 2. Patients and Methods

This descriptive study investigated children and adolescents with SCD who were admitted to the hereditary blood disease ward at the Basra Maternity and Children's Hospital for various reasons between the first of January 2012 and the end of July 2012. A total of 160 patients were recruited; their ages ranged from 9 months to 14 years.

The hospitalization event includes one or more than one admission during a period of more than 30 days from the primary admission [13]. According to this definition the total number of hospitalization events was 237.

Clinical data included age at presentation, sex, history of previous hospitalizations, the number of hospitalizations in the previous year, the number of blood transfusions in the previous year, and asthma symptoms. Disease severity was defined as ≥3 admissions and/or ≥3 blood transfusions in the previous year [13, 14].

The residence and educational level of the mother, the father, and the child (if of school age) were also reported. School attendance was assessed in school age children and was divided into regular, irregular, and left school [15].

Patient's medications like hydroxyurea (HU) and opioid, type of SCD according to the results of High Performance Liquid Chromatography (HPLC), diagnosis, and date of discharge were recorded.

Full clinical examinations were conducted for each patient, including a general examination, vital signs measurements, and a systemic examination.

Treatment, length of hospitalization, complications, outcome, and readmissions (if present) for the patient were recorded. Readmission was defined as a hospital admission occurring within 30 days of the primary admission [13].

In the Center for Hereditary Blood Diseases in Basra, we follow many strategies to reduce infections including penicillin prophylaxis for children ≤5 years old and vaccination (*Haemophilus influenzae* type b, meningococcus group C, and pneumococcal polysaccharide vaccine). It is worthy to mention that all aspects of management of these patients are free of charge. However, unfortunately we do not have neonatal screening program in Basra which enables early detection and when possible prevention of complications.

Informed consent was obtained from one or both parents for enrollment in the study. The study was approved by the Ethical Committee of the Basra Medical College.

### 2.1. Statistical Analysis

Statistical analysis was performed using SPSS program version (20) software. Data were expressed as the mean ± standard deviation (SD). Proportions were compared using cross-tabulations with chi-square tests. *t*-tests were used for quantitative comparisons and to compare the difference between two means. Comparisons between groups were made by using one-way analysis of variance (ANOVA) tests. A logistic regression analysis (Multinomial Logistic) was also performed using odds ratios (OR) with a 95% confidence interval (CI). For all tests, a *P* value of <0.05 was considered to be statistically significant.

## 3. Results

The total number of patients admitted to the general pediatric wards and the hereditary blood diseases ward at the Basra Maternity and Children's Hospital during the study period was 4140. Of these patients, 160 had SCD, with a total of 237 hospitalization events (excluding patients who were readmitted within one month), which constituted 5.75% of the total admissions during the study period.

Of the 160 admitted patients with SCD during the study period, 91 (56.88%) were male, and 69 (43.12%) were female, Table 1. Their ages ranged from 9 months to 14 years, with a mean age (±SD) of 7.97 ± 3.65 years. The majority of admitted patients with SCD had S/*β*-Thalassemia (81.9%), and 41.25% were between 5 and 10 years old.

The majority of the mothers (68.75%) and 45.61% of the fathers either were illiterate or had received primary school education. One hundred (62.5%) hospitalized patients were of school age; however, only 13% of them regularly attended school.

Acute painful crisis was the most common cause of hospitalization events (73.84%), followed by infection (9.28%), ACS (8.02%), and acute splenic sequestration crisis (ASSC) in 6.32%, Table 2. However, when considering the causes of admission in relation to number of patients both infections and ACS were found to be the second cause of admission (11.8%).

**Figure 1:**
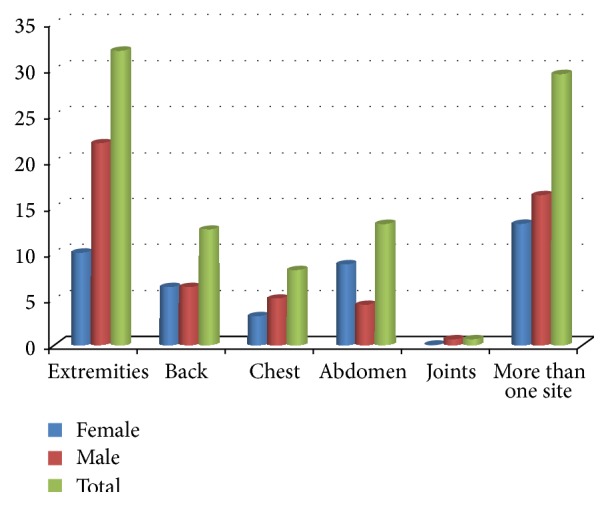
Frequency of sickle cell pain by body region and sex. *P* value = 0.178 (chi-square test).

The extremities were the most common site of pain in both sexes (33.4% overall; 10.5% of female patients and 22.9% of male patients), followed by pain in more than one site (30.7%). The least common site of pain was joint pain (0.7%), Figure 1. There were no significant differences between the sexes concerning the site of pain, *P* = 0.178.

The presence of infections was also recorded. Urine cultures for patients with urinary tract infections (UTI) revealed* E. coli* in 5 cases,* Klebsiella* spp. in 2 cases, and* Proteus* spp. in 1 case. Two patients presented with fever, and all available investigations were negative, including a blood culture.

The pattern of admissions in relation to the type of SCD was studied and it was found that there is no significant difference except for ACS which was significantly higher among patients with Hb SS disease compared to those with S/*β*-Thalassemia, Table 3.

Forty-two (26.25%) patients had no history of blood transfusion. The mean frequency of blood transfusion was 4.43 ± 0.54/year; this frequency was significantly higher among patients with S/*β*-Thalassemia (5.19 ± 0.64/year) compared to those with SCA (1.00 ± 0.21/year), *P* = 0.020.

Compared to those patients with ASSC, patients with ACS stayed for a significantly longer time at the hospital, *P* = 0.030. The mean LOS for patients on HU was significantly shorter than the LOS for patients who did not receive this drug, *P* = 0.032. However, there was no statistically significant difference in LOS in relation to age, sex, or SCD type, Table 4.

Age, sex, type of SCD, LOS, clinical events, and main treatment modalities (blood transfusion, opioid, and HU) provided to children and adolescents with SCD who were readmitted compared to those not readmitted were evaluated. The rate of readmissions among hospitalized patients was 23.1%. The mean LOS for readmitted patients was significantly longer than for patients who were not readmitted, *P* = 0.022, Table 5. A history of ≥3 hospitalization in the previous year, asthma symptoms, and opioid use were significant risk factors for readmission, *P* < 0.05. Patients on HU were less likely to be readmitted, *P* = 0.006, Table 6.

## 4. Discussion

There is still a high utilization of medical resources by patients with SCD despite the reductions in morbidity and mortality associated with early screening and the use of prophylactic antibiotics. Interventions directed at the prevention of SCD complications and hospitalizations may reduce the significant economic burden of the disease [16].

In this study, the hospitalization of patients with SCD constituted about 6% of the total number of hospitalizations in the pediatric wards, excluding patients who were admitted to the Emergency Unit and discharged.

The current study showed that approximately two-thirds of school-aged, hospitalized patients have irregular school attendance and approximately one-fourth of hospitalized patients have left school, most likely because of their illness. Shapiro et al. found that, in the USA, approximately half of school absences of SD patients are associated with SCD-related pain. Other causes of school absences include minor infections, clinic visits, and other medical problems associated with SCD. In addition, families may perceive their children as vulnerable and keep them out of school for problems that would not interfere with school attendance for most children. SCD-related pain and illness also have been shown to affect the psychosocial function and thus the school attendance of these patients [17].

Acute painful crisis was the most common cause of in-patient hospitalizations of SCD patients in this study. This finding supports results reported by Akar and Adekile in Kuwait (63.2%) [18], Jaiyesimi et al. in Oman (83%) [19], and Brown et al. in Nigeria (61.5%) [7].

Frequent acute painful crises requiring hospitalization are one of the characteristic features of SCD [20]. Individualized pain management in the emergency department is effective in improving the management quality of these crises and is associated with a high level of patient satisfaction and decreases in preventable hospitalizations [12, 20, 21].

In this study, the most common site of pain for both sexes was in the extremities. Similarly, Jaiyesimi et al. in Oman reported that 45% of hospitalized patients with SCD had pain in their extremities [19], and Fosdal and Wojner-Alexandrov in the USA found that the extremities were the most common site of pain. However, Fosdal and Wojner-Alexandrov also found that female patients were the most affected, in contrast with our findings [22].

Infections were found to be the second-leading cause of hospitalization among children and adolescents in Basra, followed by ACS then ASSC. Although infections contributed to a considerable percentage of in-patient hospitalizations, an earlier study in Basra reported a higher rate of infections (21%) among hospitalized children with SCD [23] compared to this study.

Despite the wide use of penicillin prophylaxis, the combination of suboptimal compliance and resistance to penicillin prophylaxis, nonvaccine serotypes of* S. pneumoniae*, and hyposplenism can all explain why children with SCD are still at an increased risk of bacterial infections [24].

ACS was an important cause of hospitalization among both types of SCD in this study. However, it was more common among patients with SCA compared to those with S/*β*-Thalassemia. This is consistent with research conducted by Hawasawi et al. in Saudi Arabia, who found that ACS was the third-leading cause of hospitalization events among patients with SCD [25], and by Tarer et al. in Guadeloupe, who found it to be the second-leading cause of hospitalization [26].

The LOS among hospitalized patients with SCD in relation to age and sex was not significantly different in Basra. Raphael et al. in the USA found that, after controlling for other factors, older age was the only sociodemographic variable associated with longer LOS [27]. In addition, there was no significant difference in the LOS for patients with both types of SCD, which is consistent with findings by Fosdal and Wojner-Alexandrov in the USA [22].

The mean LOS was longer for patients with ACS than for patients with ASSC. A similar finding was reported by Akar and Adekile in Kuwait, where the LOS for patients with ACS was 5.6 ± 3.3 days, and it was 3.2 ± 2.4 days for ASSC [18].

In this study, the LOS at the hospital was significantly shorter for SCD patients who were on HU compared with those who were not. Patients on HU were also less likely to be readmitted within 30 days. This can be attributed to the fact that HU improves hematological parameters and decreases the SCD-related complications (mainly acute painful crises), the required hospitalizations, and the LOS [28, 29].

Of all the patients with SCD admitted to the hospital, 23.1% were readmitted within 30 days. This result is higher than that reported by Sobota et al. in the USA (17%) [30].

The readmission rate was significantly higher among patients with ≥3 hospitalizations in the previous year and among patients with asthma symptoms. These results are similar to those reported by Frei-Jones et al. in the USA [13] but in contrast to those reported by Sobota et al. in the USA, who found that asthma was not a risk factor for readmission [30].

Asthma is associated with an increase in SCD-related morbidity. Children with SCA and a clinical diagnosis of asthma had nearly twice as many episodes of ACS and more frequent painful episodes, which are 2 leading causes of hospitalization. Based on the pathogenesis of asthma and the prevalence of airway obstruction and airway liability, ventilation-perfusion mismatching may cause local tissue hypoxia, promote increased sickling of red blood cells, and initiate an ACS or a vasoocclusive pain episode [31].

The current study reported that patients with SCD who received opioids during their hospitalization were more likely to be readmitted, while patients who were on HU were less likely to be readmitted. This result could be because an increase in the levels of acute phase reactants that bind to opioids makes them unavailable to induce pain relief, the development of tolerance to opioids, or changes at the opioid receptor sites [21]. However, Loureiro et al. in Brazil did not find such an association; the only risk factors for readmission found in that study were previous vasoocclusive crises and renal failure [32].

Most patients were discharged in good health, and no deaths were reported in this study. This result could be related to the severity of the disease or to the short study period.

The main limitation of this study is its short duration. A longer duration may have revealed other causes of morbidity, and it would have enabled the assessment of mortality among these patients.

It can be concluded from this study that although acute painful crises were the most common cause of hospitalization and readmission among patients with SCD, infections were still reported in a significant proportion of patients with SCD. In addition, there was a relatively high rate of readmission, and the use of HU was associated with shorter LOS and fewer hospital readmissions.

## Figures and Tables

**Table 1:** Selected sociodemographic characteristics of patients.

Variable	SCA		S/*β*-Thalassemia		Total	
	Number	%	Number	%	Number	%
Age (years)						
≤5	10	6.24	34	21.26	44	27.5
>5–10	14	8.74	52	32.51	66	41.25
>10–14	5	3.12	45	28.13	50	31.25
Sex						
Male	14	8.74	77	48.14	91	56.88
Female	15	9.36	54	33.76	69	43.12
Residence						
Urban	13	8.11	70	43.76	83	51.87
Rural	16	9.99	61	38.14	77	48.13
Educational level of father						
Illiterate	1	0.62	10	6.25	11	6.87
Primary	15	9.36	47	29.38	62	38.74
Secondary	6	3.75	54	33.75	60	37.5
High education	7	5.45	20	12.5	27	16.95
Educational level of mother						
Illiterate	2	1.25	17	10.63	19	11.88
Primary	18	11.23	73	45.64	91	56.87
Secondary	7	4.37	30	18.75	37	23.12
High education	2	1.25	11	6.88	13	8.13
Total	29	18.1	131	81.9	160	100
School attendance for children (number, 100)						
Regular	2	2	11	11	13	13
Irregular	15	15	49	49	64	64
Left	1	1	22	22	23	23

**Table 2:** Causes of hospital admission.

Cause	Admitted patients		Hospitalization events	
	Number	%	Number	%
Acute painful crisis	104	65	175	73.84
ACS/pneumonia	19	11.88	19	8.02
ASSC	13	8.13	15	6.32
Infection				
Urinary tract infection	8	5	10	4.23
Hepatitis A	4	2.5	4	1.69
Gastroenteritis	3	1.88	4	1.69
Fever	2	1.25	2	0.84
Cervical lymphadenitis	2	1.25	2	0.84
Bleeding due to hypersplenism	2	1.25	3	1.27
AVN	1	0.63	1	0.42
Stroke	1	0.63	1	0.42
Neuroblastoma	1	0.63	1	0.42

Total	160	100	237	100

ACS: acute chest syndrome, ASSC: acute splenic sequestration crises, and AVN: avascular necrosis.

**Table 3:** Causes of admission in relation to type of SCD.

Causes of admission	SCA		S/*β*-Thalassemia		Total	
	Number	%	Number	%	Number	%
Acute painful crisis	16	55.17	88	67.18	104	65
ACS^*∗*^	8	27.59	11	8.4	19	11.88
ASSC	2	6.89	11	8.4	13	8.13
Infection						
UTI			8	6.11	8	5
Hepatitis A	1	3.45	3	2.29	4	2.5
Gastroenteritis	1	3.45	2	1.53	3	1.88
Fever			2	1.53	2	1.25
Cervical lymphadenitis	1	3.45	1	0.76	2	1.25
Bleeding due to hypersplenism			2	1.53	2	1.25
AVN			1	0.76	1	0.63
Stroke			1	0.76	1	0.63
Neuroblastoma			1	0.76	1	0.63

Total	29	100	131	100	160	100

^*∗*^ *P* value = 0.007 (chi-square).

ACS: acute chest syndrome, ASSC: acute splenic sequestration crises, AVN: avascular necrosis, and UTI: urinary tract infection.

**Table 4:** Length of hospital stay in relation to selected variables among patients with SCD.

Variable	LOS (mean ± SD)	*P* value
Age (year)		
≤5	3.81 ± 2.44	0.350
>5–10	4.63 ± 3.44	
>10	4.42 ± 2.25	
Sex		
Male	4.18 ± 2.62	0.421
Female	4.55 ± 3.14	
Type of SCD		
SCA	4.48 ± 2.16	0.881
S/*β*-Thalassemia	4.31 ± 2.98	
HU		
Not received	4.59 ± 2.86	0.032
Received	3.41 ± 2.64	
Final diagnosis		
Acute painful crisis	4.10 ± 2.75	0.030^*∗*^
ACS/pneumonia	5.10 ± 2.44	
ASSC	2.61 ± 0.96	

*P* value calculated by ANOVA test for age and final diagnosis and by *t*-test for other variables.

^*∗*^Significantly different between ACS and splenic sequestration in relation to length of stay.

ACS: acute chest syndrome, ASSC: acute splenic sequestration crises, HU: hydroxyurea, LOS: length of stay, SCD: sickle cell disease, and SCA: sickle cell anemia.

**Table 5:** Readmission among hospitalized patients with SCD.

Variable	Readmission	No readmission	*P* value
	Number (37)	Number (123)	

Mean age (year) ± SD^*∗*^	8.55 ± 3.53	7.80 ± 3.68	0.272
Mean LOS ± SD^*∗*^	5.45 ± 3.45	4.01 ± 2.56	0.022

	Number (%)	Number (%)	

Sex			
Male	19 (51.35)	72 (58.54)	0.431
Female	18 (48.65)	51 (41.46)	
Type of SCD			
SCA	6 (16.22)	23 (18.70)	0.732
S/*β*-Thalassemia	31 (83.78)	100 (81.30)	
Final diagnosis			
Acute painful crisis	21 (56.76)	83 (67.48)	0.572
ACS	2 (5.40)	17 (13.82)	
ASSC	3 (8.11)	10 (8.13)	
Hospitalization in previous year			
No	2 (5.41)	42 (34.15)	0.000
<3	9 (24.32)	41 (33.33)	
≥3	26 (70.27)	40 (32.52)	
Blood transfusion in previous year			
No	5 (13.51)	37 (30.10)	0.027
<3	13 (35.14)	50 (40.70)	
≥3	19 (51.35)	36 (29.30)	
Blood transfusion during admission			
Not received	19 (51.35)	64 (52.03)	0.942
Received	18 (48.65)	59 (47.97)	
Asthma symptom			
No	16 (43.24)	88 (71.54)	0.002
Yes	21 (56.75)	35 (28.46)	
Opioid received			
No	19 (51.35)	113 (91.87)	0.000
Yes	18 (48.65)	10 (8.13)	
HU received			
No	33 (89.19)	93 (75.61)	0.077
Yes	4 (10.81)	30 (24.39)	

^*∗*^ *P* values for age and LOS were assessed by *t*-test and for other variables by chi-square.

ACS: acute chest syndrome, ASSC: acute splenic sequestration crises, HU: hydroxyurea, LOS: length of stay, SCD: sickle cell disease, and SCA: sickle cell anemia.

**Table 6:** Logistic regression analysis of different variables with readmission.

Variable	OR	95% (CI)		*P* value
		Lower	Upper	
Age (years)	0.674	0.105	4.327	0.911
LOS	1.073	0.048	24.032	0.688
Sex	0.352	0.087	1.444	0.141
Type of SCD	0.431	0.076	2.445	0.350
Acute painful crisis	1.608	0.150	17.209	0.221
Hospitalization in previous year ≥ 3	9.352	2.011	43.490	0.001
Blood transfusion in previous year ≥ 3	3.325	0.477	23.163	0.393
Asthma symptoms	4.225	1.125	15.862	0.028
Opioid received	6.588	1.104	30.336	0.000
HU received	0.082	0.010	0.663	0.006

HU: hydroxyurea, LOS: length of stay, and SCD: sickle cell disease.

## References

[1] Cançado R. D. (2012). Sickle cell disease: looking back but towards the future. *Revista Brasileira de Hematologia e Hemoterapia*.

[2] Hassan M. K., Taha J. Y., Al-Naama L. M., Widad N. M., Jasim S. N. (2003). Frequency of haemoglobinopathies and glucose-6-phosphate dehydrogenase deficiency in Basra. *Eastern Mediterranean Health Journal*.

[3] Brandow A. M., Liem R. I. (2011). Sickle cell disease in the emergency department: atypical complications and management. *Clinical Pediatric Emergency Medicine*.

[4] De Baun M. R., Frei-Jones M., Vichinsky E., Behrman E. R., Kliegman R. M., Jenson H. B. (2011). Hemglobinobathies. *Nelson TextBook of Pediatrics*.

[5] Colombatti R., Montanaro M., Guasti F. (2012). Comprehensive care for sickle cell disease immigrant patients: a reproducible model achieving high adherence to minimum standards of care. *Pediatric Blood and Cancer*.

[6] Quinn C. T., Rogers Z. R., McCavit T. L., Buchanan G. R. (2010). Improved survival of children and adolescents with sickle cell disease. *Blood*.

[7] Brown B. J., Jacob N. E., Lagunju I. A., Jarrett O. O. (2013). Morbidity and mortality pattern in hospitalized children with sickle cell disorders at the University College Hospital, Ibadan, Nigeria. *Nigerian Journal of Paediatrics*.

[8] Aljuburi G., Laverty A. A., Green S. A., Phekoo K. J., Bell D., Majeed A. (2013). Socio-economic deprivation and risk of emergency readmission and inpatient mortality in people with sickle cell disease in England: observational study. *Journal of Public Health*.

[9] Booth C., Inusa B., Obaro S. K. (2010). Infection in sickle cell disease: a review. *International Journal of Infectious Diseases*.

[10] Ikefuna A. N., Emodi I. J. (2007). Hospital admission of patients with sickle cell anaemia pattern and outcome in Enugu area of Nigeria. *Nigerian Journal of Clinical Practice*.

[11] Lanskowsky P., Arkin S., Atlas M., Aygun B., Friedman D., Lanzkowsky P. (2011). Hemoglobin defects, sickle cell disease. *Manual of Pediatric Hematology and Oncology*.

[12] Krishnamurti L., Smith-Packard B., Gupta A., Campbell M., Gunawardena S., Saladino R. (2014). Impact of individualized pain plan on the emergency management of children with sickle cell disease. *Pediatric Blood and Cancer*.

[13] Frei-Jones M. J., Field J. J., DeBaun M. R. (2009). Risk factors for hospital readmission within 30 days: a new quality measure for children with sickle cell disease. *Pediatric Blood and Cancer*.

[14] Jain D., Italia K., Sarathi V., Ghoshand K., Colah R. (2012). Sickle cell anemia from central India: a retrospective analysis. *Indian Pediatrics*.

[15] Al Arrayed S. S., Haites N. (1995). Features of sickle cell disease in Bahrain. *Eastern Mediterranean Health Journal*.

[16] Kauf T. L., Coates T. D., Huazhi L., Mody-Patel N., Hartzema A. G. (2009). The cost of health care for children and adults with sickle cell disease. *American Journal of Hematology*.

[17] Shapiro B. S., Dinges D. F., Orne E. C. (1995). Home management of sickle cell-related pain in children and adolescents: natural history and impact on school attendance. *Pain*.

[18] Akar N. A., Adekile A. (2008). Ten-year review of hospital admissions among children with sickle cell disease in Kuwait. *Medical Principles and Practice*.

[19] Jaiyesimi F., Pandey R., Bux D., Sreekrishna Y., Zaki F., Krishnamoorthy N. (2002). Sickle cell morbidity profile in Omani children. *Annals of Tropical Paediatrics*.

[20] Lamarre Y., Romana M., Waltz X. (2012). Hemorheological risk factors of acute chest syndrome and painful vaso-occlusive crisis in children with sickle cell disease. *Haematologica*.

[21] Ballas S. K. (2007). Current issues in sickle cell pain and its management. *Hematology/the Education Program of the American Society of Hematology*.

[22] Fosdal M. B., Wojner-Alexandrov A. W. (2007). Events of hospitalization among children with sickle cell disease. *Journal of Pediatric Nursing*.

[23] Ali I. A., Hassan M. K. (1999). Sickle cell syndrome in children in Basrah. *Medical Journal of Tikrit*.

[24] Chakravorty S., Williams T. N. (2015). Sickle cell disease: a neglected chronic disease of increasing global health importance. *Archives of Disease in Childhood*.

[25] Hawasawi Z. M., Nabi G., Al Magamci M. S. F., Awad K. S. (1998). Sickle cell disease in childhood in Madina. *Annals of Saudi Medicine*.

[26] Tarer V., Etienne-Julan M., Diara J.-P. (2006). Sickle cell anemia in Guadeloupean children: pattern and prevalence of acute clinical events. *European Journal of Haematology*.

[27] Raphael J. L., Mueller B. U., Kowalkowski M. A., Oyeku S. O. (2012). Shorter hospitalization trends among children with sickle cell disease. *Pediatric Blood and Cancer*.

[28] Ballas S. K., Bauserman R. L., McCarthy W. F., Castro O. L., Smith W. R., Waclawiw M. A. (2010). Hydroxyurea and acute painful crises in sickle cell anemia: effects on hospital length of stay and opioid utilization during hospitalization, outpatient acute care contacts, and at home. *Journal of Pain and Symptom Management*.

[29] Mulaku M., Opiyo N., Karumbi J., Kitonyi G., Thoithi G., English M. (2013). Evidence review of hydroxyurea for the prevention of sickle cell complications in low-income countries. *Archives of Disease in Childhood*.

[30] Sobota A., Graham D. A., Neufeld E. J., Heeney M. M. (2012). Thirty-day readmission rates following hospitalization for pediatric sickle cell crisis at freestanding children's hospitals: risk factors and hospital variation. *Pediatric Blood & Cancer*.

[31] Boyd J. H., Macklin E. A., Strunk R. C., DeBaun M. R. (2006). Asthma is associated with acute chest syndrome and pain in children with sickle cell anemia. *Blood*.

[32] Loureiro M. M., Rozenfeld S., Carvalho M. S., Portugal R. D. (2009). Factors associated with hospital readmission in sickle cell disease. *BMC Blood Disorders*.

